# Supplementary material for: Pyruvate Dehydrogenase Kinase 1 inhibition mediated oxidative phosphorylation enhancement in cartilage promotes osteoarthritis progression
Source: BMC Musculoskelet Disord. 2023 Jul 20;24:597. doi: 10.1186/s12891-023-06585-6 (PMC10357736; doi:10.1186/s12891-023-06585-6)
Supplement: Supplementary file 1 — Additional file 1. [file 12891_2023_6585_MOESM1_ESM.zip › BP.Gsea.1653623667859/GOBP_REGULATION_OF_CELLULAR_RESPONSE_TO_HEAT.html]

Details for gene set GOBP\_REGULATION\_OF\_CELLULAR\_RESPONSE\_TO\_HEAT[GSEA]

|  || Dataset | 55457\_collapsed\_to\_symbols.cls.cls #OA\_versus\_normal.cls.cls #OA\_versus\_normal\_repos |
| Phenotype | cls.cls#OA\_versus\_normal\_repos |
| Upregulated in class | normal |
| GeneSet | GOBP\_REGULATION\_OF\_CELLULAR\_RESPONSE\_TO\_HEAT |
| Enrichment Score (ES) | -0.62619615 |
| Normalized Enrichment Score (NES) | -1.8376842 |
| Nominal p-value | 0.0017699115 |
| FDR q-value | 0.07516241 |
| FWER p-Value | 0.934 |
Table: GSEA Results Summary

  

Fig 1: Enrichment plot: GOBP\_REGULATION\_OF\_CELLULAR\_RESPONSE\_TO\_HEAT      
 Profile of the Running ES Score & Positions of GeneSet Members on the Rank Ordered List

  

| SYMBOL | TITLE | RANK IN GENE LIST | RANK METRIC SCORE | RUNNING ES | CORE ENRICHMENT || 1 | MAPT | microtubule associated protein tau [Source:HGNC Symbol;Acc:HGNC:6893] | 3976 | 0.158 | -0.2822 | No |
| 2 | CD34 | CD34 molecule [Source:HGNC Symbol;Acc:HGNC:1662] | 4341 | 0.125 | -0.2896 | No |
| 3 | ATM | ATM serine/threonine kinase [Source:HGNC Symbol;Acc:HGNC:795] | 6179 | -0.019 | -0.4291 | No |
| 4 | IER5 | immediate early response 5 [Source:HGNC Symbol;Acc:HGNC:5393] | 7334 | -0.105 | -0.5011 | No |
| 5 | SIRT1 | sirtuin 1 [Source:HGNC Symbol;Acc:HGNC:14929] | 8947 | -0.227 | -0.5882 | Yes |
| 6 | DNAJB1 | DnaJ heat shock protein family (Hsp40) member B1 [Source:HGNC Symbol;Acc:HGNC:5270] | 9380 | -0.262 | -0.5778 | Yes |
| 7 | DNAJC7 | DnaJ heat shock protein family (Hsp40) member C7 [Source:HGNC Symbol;Acc:HGNC:12392] | 9626 | -0.285 | -0.5491 | Yes |
| 8 | DNAJC2 | DnaJ heat shock protein family (Hsp40) member C2 [Source:HGNC Symbol;Acc:HGNC:13192] | 10081 | -0.329 | -0.5294 | Yes |
| 9 | DNAJB6 | DnaJ heat shock protein family (Hsp40) member B6 [Source:HGNC Symbol;Acc:HGNC:14888] | 10702 | -0.394 | -0.5117 | Yes |
| 10 | CREBBP | CREB binding protein [Source:HGNC Symbol;Acc:HGNC:2348] | 10881 | -0.414 | -0.4562 | Yes |
| 11 | GSK3B | glycogen synthase kinase 3 beta [Source:HGNC Symbol;Acc:HGNC:4617] | 11157 | -0.451 | -0.4021 | Yes |
| 12 | HSF1 | heat shock transcription factor 1 [Source:HGNC Symbol;Acc:HGNC:5224] | 11189 | -0.456 | -0.3283 | Yes |
| 13 | ATR | ATR serine/threonine kinase [Source:HGNC Symbol;Acc:HGNC:882] | 11231 | -0.461 | -0.2544 | Yes |
| 14 | CHORDC1 | cysteine and histidine rich domain containing 1 [Source:HGNC Symbol;Acc:HGNC:14525] | 11689 | -0.531 | -0.2012 | Yes |
| 15 | EP300 | E1A binding protein p300 [Source:HGNC Symbol;Acc:HGNC:3373] | 11741 | -0.540 | -0.1149 | Yes |
| 16 | MTOR | mechanistic target of rapamycin kinase [Source:HGNC Symbol;Acc:HGNC:3942] | 12047 | -0.605 | -0.0374 | Yes |
| 17 | MAPKAPK2 | MAPK activated protein kinase 2 [Source:HGNC Symbol;Acc:HGNC:6887] | 12094 | -0.619 | 0.0626 | Yes |
Table: GSEA details [plain text format]

  

Fig 2: GOBP\_REGULATION\_OF\_CELLULAR\_RESPONSE\_TO\_HEAT      
 Blue-Pink O' Gram in the Space of the Analyzed GeneSet

  

Fig 3: GOBP\_REGULATION\_OF\_CELLULAR\_RESPONSE\_TO\_HEAT: Random ES distribution      
 Gene set null distribution of ES for **GOBP\_REGULATION\_OF\_CELLULAR\_RESPONSE\_TO\_HEAT**
